# Supplementary material for: Comparison by Life-Cycle Assessment of Alternative Processes for Carvone and Verbenone Production
Source: Molecules. 2022 Aug 26;27(17):5479. doi: 10.3390/molecules27175479 (PMC9457752; doi:10.3390/molecules27175479)
Supplement: Supplementary file 1 [file molecules-27-05479-s001.zip › molecules-1775286-supplementary.pdf]

Supplementary Materials

Comparison by Life-Cycle Assessment of Alternative Processes for Carvone and Verbenone Production

Jaime-Andrés Becerra *, Juan-Miguel González and Aída-Luz Villa *

Environmental Catalysis research group, Chemical Engineering Department, Engineering Faculty, Universidad de Antioquia UdeA, Calle 70 No. 52-21, Medellín, Colombia

* Correspondence: aida.villa@udea.edu.co; Tel.: (+57 4) 2196605, (+57 4) 2198535, (A.-L.V.), jaime.becerra@udea.edu.co (J.-A.B.)

1. Details of the life cycle inventory analysis.

S.1. Orange essential oil extraction

Owing to the lack of information about orange essential oil extraction in SimaPro databases, a plant simulation was implemented in the software Aspen Plus, for the production of orange oil from raw oranges, based on the information reported by Becalli et al [1], **Figure S1**. The process begins with an extraction stage, in which, juice pulp and a water-essential oil emulsion are obtained. Water is added to the water-oil emulsion to avoid volatilization of the oil. Subsequently, in an oil refining stage, solid impurities are removed by filtration and water is removed by centrifugation. Finally, the oil is stored at up to 2°C for conservation purposes.

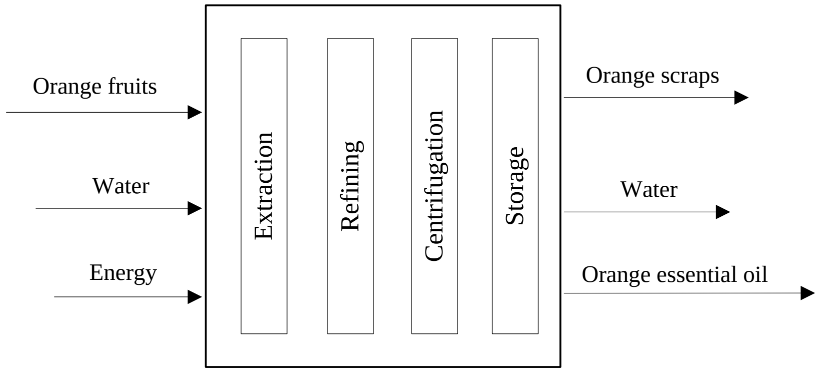

Figure S1. Diagram for the production of orange essential oil.

S.1.1. Separation of d-limonene and α-pinene

The purification process of α-pinene and d-limonene from the essential oils was carried out based on preliminary technical and economic feasibility analysis of the fractional vacuum distillation in T-102 [2]. Since α-pinene and d-limonene are not available in SimaPro databases, their impacts were considered as being a component resulting from the turpentine and orange-peel oils separation, respectively.

S.2. TBHP synthesis

TBHP synthesis was implemented in the software Aspen Plus, based on patented information regarding the oxidation of isobutane with oxygen at 800 psi and 405 K. Isobutane synthesis by the isomerization of butane at 293 K and 44.1 psi in the presence of HCl and $AlCl_3$ was also simulated in Aspen Plus.

S.3. $FePcCl_{16}$-NH_2-SiO_2 catalyst preparation

The laboratory scale synthesis of the catalyst $FePcCl_{16}$-NH_2-SiO_2 can be divided into three steps: preparation of the metallic complex or active phase $FePcCl_{16}$, synthesis of the modified support NH_2-SiO_2 and the immobilization of the metal complex on the modified support based on literature procedures [3,4]. All the data used for the implementation of the LCIA is presented in **Table S1.**

S.3.1. Step 1, synthesis of active phase complex $FePcCl_{16}$

- *$FePcCl_{16}$*

The hexadecachlorinated iron phthalocyanine complex $FePcCl_{16}$ is synthesized using a mixture of urea, tetrachlorophthalic anhydride, $FeCl_2.4H_2O$, ammonium heptamolybdate tetrahydrate catalyst and nitrobenzene as solvent at 463 K according with some reports [3]. Further procedures for neutralization, purification and drying steps include additional energy requirements and the use of different solvents such as ethanol, water, aqueous solutions of HCl and NaOH, H_2SO_4, acetone and dichloromethane. The implementation of the process considers total conversion of tetrachlorophthalic anhydride, stoichiometric byproducts from **Figure S2a**, and total recovering of catalyst and solvents during separation stages. In addition, the production of tetrachlorophthalic anhydride and ammonium heptamolybdate were addressed since the processes were not available in the selected databases.

- *Tetrachlorophthalic anhydride*

A methodology for the production of tetrachlorophthalic anhydride from phthalic anhydride and chlorine in the presence of molybdenum-based catalysts in **Figure S2c** was patented by [5]. In a

general procedure, metallic molybdenum powder was added to molten phthalic anhydride in a tubular glass reactor. Chlorine gas was fluxed and the temperature was gradually raised from 200 to 270°C. The average chlorine conversion was 72% and the product was purified. The implementation of this process was based on 94% conversion of phthalic anhydride, total selectivity to tetrachlorophthalic anhydride with stoichiometric HCl as the only co-product, and only energy requirement for heating in the reaction since the purification data were not reported.

- *$(NH_4)_6Mo_7O_{24}$*

The production of ammonium heptamolybdate tetrahydrate from the treatment of MoO_3 with ammonia by **Figure S2d** was patented by [6]. In a general procedure, reroasted molybdic oxide feedstock containing 90-98% MoO_3 is treated with aqueous and then ammoniacal solutions for leaching. The resulting solution containing dissolved ammonium molybdate compounds is saturated with gaseous CO_2 and crystallized to ammonium heptamolybdate with a yield of 45% of the molybdenum content originally present in the feed material. The implementation based on total conversion of ammoniacal leaching solution containing molybdenum trioxide into $(NH_4)_6Mo_7O_{24}$, however in previous separation stages amounts of MoO_3 have already been lost.

S.3.2. Step 2, synthesis of modified support $FePcCl_{16}$

- *NH_2-SiO_2*

Fuming silica-based support was modified with 3-aminopropyltriethoxysilane (APTES) following the procedure reported by [4], as shown in **Figure S2b**. In a general procedure, APTES was added to a suspension of thermal activated (423 K, vacuum) fuming amorphous silica SiO_2 in *m*-xylene. The final mixture was heated to 423K, refluxed under argon, filtered, washed with acetone, and dried at 353 K under vacuum. The implementation of the process considers total incorporation of APTES on the activated silica and the ethanol as the only co-product obtained as a molar 3:1 ratio respect to the amount of APTES incorporated in the support, whose synthesis procedure was also addressed.

- *APTES*

The implementation of the process for the production of 3-aminopropyl triethoxysilane was based on the synthesis procedure from triethoxysilane (TES) and allylamine patented by [7], as it is shown in **Figure S2e**. In a general procedure TES, solvent *p*-xylene and tricarbonyl-bis(triphenylphosphine) ruthenium catalyst (0.1 mol% of Ru catalyst per mol of TES) were charged to a reactor, heated at 110°C and allylamine slowly added. After the reaction completion 62% yield of γ-aminopropyl triethoxysilane (APTES) and 0.52% yield of β-aminopropyl triethoxysilane were obtained. It has been reported that

in this route TES addition is slightly inhibited by Pt catalysts and produces too much by-product β-adduct (resulting from internal addition, 10-25%) causing problems in many applications. Ruthenium and rhodium phosphine complexes increase the overall yield of the desired γ product in >60-80% [7,8]. The implementation of the process considers 62% yield with total selectivity to γ-adduct APTES, using only the active metallic Rh phase (available in the Ecoinvent database) as catalyst instead of Ru complex, and processes for the production of allylamine and TES were also implemented.

- *Triethoxysilane*

The production of TES from silicon and ethanol in the presence of copper catalysts in **Figure S2f** was patented by [9]. In a general procedure, a solid mixture of silicon powder with copper oxides (1:1 CuO and Cu_2O) previously activated via microwave heating at 270 °C is placed in an autoclave with a thermal oil and the Raney copper catalyst, then mixed to homogeneously disperse the silicon powder, heated at 230°C with ethanol addition. TES with 95% selectivity and tetraethoxysilane (TEOS) are obtained as distillate products for an 83% silicon conversion. The collected TES distillate was efficiently fractionated in a rectification tower until ≥99% purity. For the implementation of the process, stoichiometric amounts of ethanol and Si were considered in the feed and a generic thermal oil available in the Ecoinvent database.

- *Allylamine*

The implementation of the process for the production of allylamine from ammonia and allyl chloride was based on the synthesis methodology patented by [10], from the reaction shown in **Figure S2g**. In a general procedure, allyl chloride and aqueous ammonia solution were loaded in a reaction vessel at 97 - 105°C equipped with heating, cooling and stirring. The reaction mixture was distilled to remove unreacted reagents, distillation residue neutralized with NaOH and distilled again to remove the primary allylamine with 77.1% molar yield, secondary and tertiary allylamines with 6.0% and 1.9% yield respectively were further recovered by steam distillation. It was assumed only the production of the primary allylamine since the other by-products are not fully specified in the patent, all the ammonium chloride generated in the neutralization stage with NaOH is stoichiometric transformed into NH_3, NaCl and water.

S.3.3. Step 3, immobilization of active phase complex $FePcCl_{16}$

The $FePcCl_{16}$-NH_2-SiO_2 catalyst was obtained by dissolving the $FePcCl_{16}$ complex in pyridine at room temperature. A suspension of the modified support NH_2-SiO_2 in pyridine was mixed with the complex-pyridine solution under argon flow at room temperature at 393 K. The green solid obtained after cooling was washed with acetone and dried. For the implementation of this process, it was considered a total

immobilization of the complex on the support, no generation of co-products and total solvent recovering.

Table S1. Inventory data for the processes considered in the production of the heterogeneous catalyst $FePcCl_{16}$-NH_2-SiO_2.

Materials/Energy	Input	Output (products +co-products)	Output (other emissions)
	$FePcCl_{16}$-NH_2-SiO_2		
$FePcCl_{16}$ (g)	0.18	0	0
NH_2-SiO_2 (g)	3	0	0
$FePcCl_{16}$-NH_2-SiO_2 (g)		3.18	0
Pyridine (mL)	90	0	90
Ar (mL/min)	100	0	100
Acetone (mL)	200	0	200
Electricity (kWh)	14.286	0	0
	$FePcCl_{16}$ production		
$C_8Cl_4O_3$ (g)	16	0	0
Urea (g)	11.7	0	8.34
$FeCl_2$ (g)	2.18	0	0.407
$(NH_4)_6Mo_7O_{24}$ (g)	0.0942	0	0.0942
$FePcCl_{16}$ (g)	0	15.66	
CO_2 (g)	0	0	2.463
NH_3 (g)	0	0	2.632
H_2O (g)	398.6258	0	400.6422
Nitrobenzene (mL)	50	0	50
Ethanol (mL)	50	0	50
HCl (g)	1	0	0.08855
NaOH (g)	1	0	0
NaCl (g)	0	0	1.461
H_2SO_4 (mL)	50	0	50
Dichloromethane (mL)	10	0	10
Acetone (mL)	100	0	100
Electricity (kWh)	6.348	0	0
	Tetrachlorophthalic anhydride production		
Phthalic anhydride (g)	200	0	12
Cl_2 (g)	500	0	140
Tetrachlorophthalic anhydride (g)	0	362.9	0
Mo (g)	1.4	0	1.4
HCl (g)	0	0	185.13
Electricity (kWh)	6.545	0	0
	$(NH_4)_6Mo_7O_{24}$ production		
MoO_3 (g)	147	0	41.54
NH_3 (g)	66.823	0	56.128
H_2O (g)	763.602	0	754.178

$(NH_4)_6Mo_7O_{24}$ (g)	0	66.15	55.671
CO_2 (g)	14.7	0	14.7
Electricity (kWh)	0.520	0	0
NH_2-SiO_2 production			
SiO_2 (g)	5	0	0
APTES (mL)	0.65	0	0
NH_2-SiO_2 (g)	0	5.231	0
Ethanol (g)	0	0	0.3839
m-xylene (mL)	100	0	100
Acetone (mL)	300	0	300
Ar (mL/min)	100		100
Electricity (kWh)	23.360	0	0
APTES production			
Allylamine (g)	14	0	5.321
Trietoxysilane (g)	41	0	16.025
p-Xylene (mL)	20	0	20
Rh (g)	0.025725	0	0.025725
APTES (g)	0	33.655	0
Electricity (kWh)	1.195	0	0
Allylamine production			
Allyl chloride (g)	1239.786	0	283.911
NH_3 (g)	2759.022	0	2546.293
H_2O (g)	7094.628	0	7319.6405
NaOH (g)	499.567	0	0
Allylamine (g)	0	713.0655	0
NaCl (g)	0	0	729.956
Electricity (kWh)	0.285	0	0
TES production			
Si (g)	100		17
Ethanol (g)	413.73	0	0
TES (g)	0	368.8	92.2
TEOS (g)	0	0	24
H_2 (g)	0	0	6.192
Thermal oil (mL)	400		400
CuO (g)	1.5	0	1.5
Cu_2O (g)	1.5	0	1.5
Raney Cu (g)	2	0	2
Electricity (kWh)	1.759	0	0

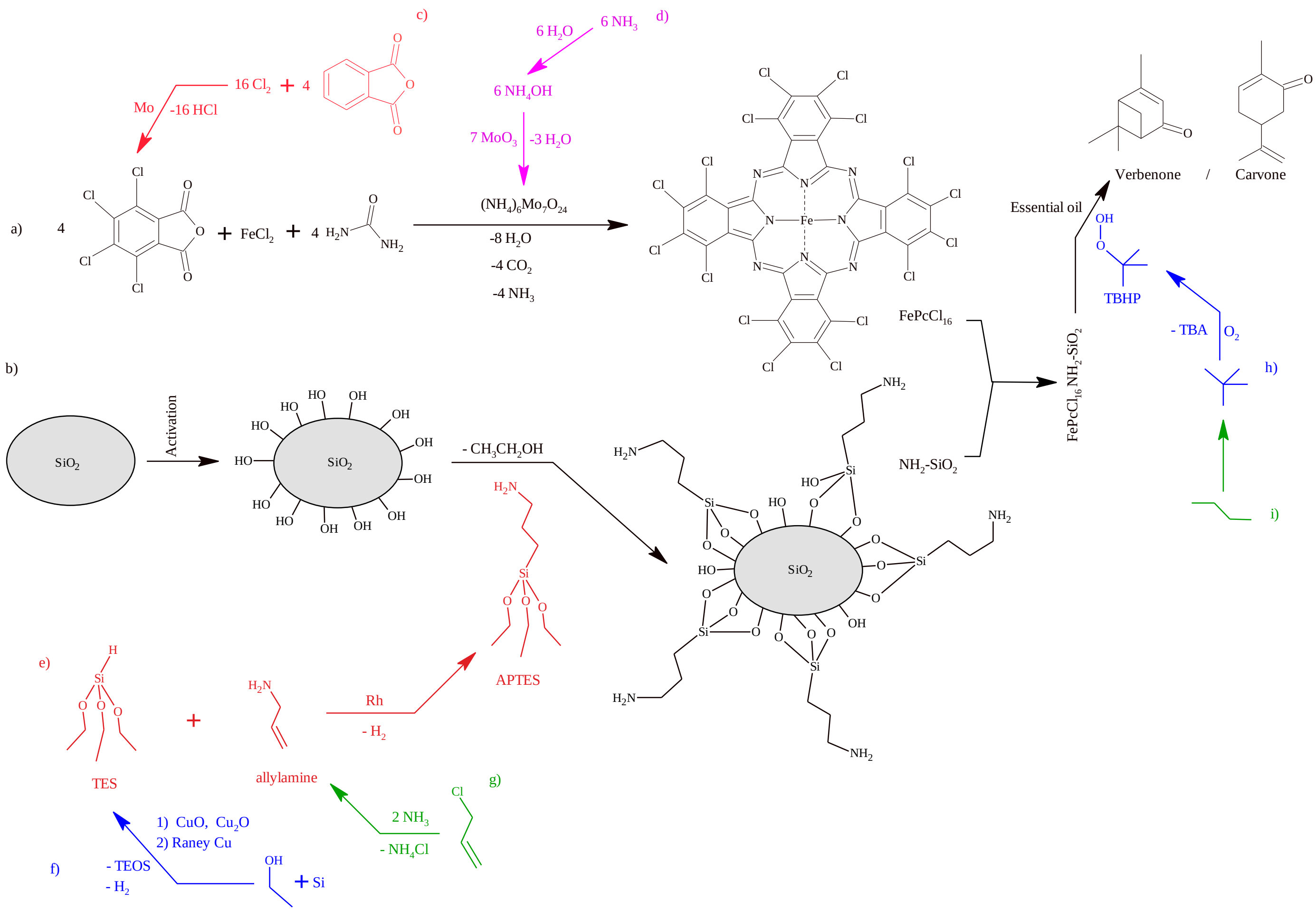

Figure S2. Chemical routes considered for the implementation of the LCI for the batch production of verbenone and carvone from turpentine and orange oils oxidation with TBHP and $FePcCl_{16}$-NH_2-SiO_2.

Table S2 to **Table S8** contain the mass and energy input/output data for the implementation of the inventory in the process for production of verbenone and carvone.

Table S2. Inventory data for the processes considered in the production of verbenone in the conditioning zone.

	**Input (kg)**	**Output (kg)**	
**Scenario**	**SII-purified-oils**		
**Compound**	**T-102**	**T-102 D**	**T-102 B**
α-Pinene	286.7188	281.6019	0
b-Pinene	42.7288	4.6793	0.0004
d-3-Carene	16.7748	0.0581	0.0002
d-Limonene	8.8547	0	0.0112
Camphene	4.8953	4.3015	0

Table S3. Inventory data for the processes considered in the production of verbenone in the reaction zone.

	Input (kg)		Output (kg)	
**Scenario**	**SI-raw-oils**	**SII-purified-oils**	**SI-raw-oils**	**SII-purified-oils**
**Compounds**	**R-101**			
Catalyst	12.5	12.5	12.5	12.5
α-Pinene	194.9036	194.9034	95.633	97.2747
TBHP	465.8383	465.8385	116.5945	118.8844
β-Pinene	29.0493	3.2387	10.2904	1.2159
d-3-Carene	11.4044	0.0402	2.518	0.009
d-Limonene	6.0199	0	2.1173	0
Camphene	3.3283	8.7699	2.4647	6.46
Verbenone	0	0	27.2326	27.6302
Verbenol	0	0	5.8107	5.8955
α-pineno epoxyde	0	0	12.8565	13.0441
t-Butanol	0	0	316.7432	321.3677
Water	203.48	203.48	245.1095	248.9453
Acetone	751.6248	751.6248	751.6247	751.6249
Linalool	0	0	8.8645	0
2-Methylenebicyclo[2.1.1]_hexane	0	0	0	2.2505
cis-Pineno-3-ol	0	0	0	0.8226
1-Cyclohexene-1-acetaldehydo,α,2-dimethyl	0	0	0	2.2507
Pinocarvone	0	0	5.5238	0.6584
Myrtenal	0	0	5.2372	0.827
Menthene	0	0	4.0001	0
Isocamphane	0	0	2.9748	3.9815
α-Fenchene	0	0	2.3406	3.1107
α-Farnesene	0	0	10.3479	0
Lynalyl isobutirate	0	0	37.077	21.7246

Table S4. Inventory data for the processes considered in the production of verbenone in the separation zone.

	Input (kg)		Output (kg)									
**Scenario**	**SI-raw-oils**	**SII-purified-oils**	**SI-raw-oils**					**SII-purified-oils**				
**Compound**	**P-101**		**F-101 S**	**D-101 D**	**T-101 D1**	**T-101 D2**	**T-101 B**	**F-101 S**	**D-101**	**T-101 D1**	**T-101 D2**	**T-101 B**
Catalyst	12.5	12.5	12.5	0	0	0	0	12.5	0	0	0	0
α-Pinene	95.633	97.275	0.752	88.308	6.545	0	0	0.935	94.864	1.068	0.033	0
TBHP	116.594	118.884	1.28	105.673	9.758	0	0	1.051	116.877	0.652	0	0

b-Pinene	10.29	1.216	0.303	8.875	1.541	0.001	0	0	1.349	0.04	0.005	0
d-3-Carene	2.518	0.009	0.248	2.219	0.427	0.001	0	0	0.008	0.001	0	0
d-Limonene	2.117	0	0	1.331	0.628	0.011	0	0	0	0	0	0
Camphene	2.465	6.46	0.195	1.331	1.103	0.001	0	0.098	4.945	0.98	0.085	0
Verbenone	27.233	27.63	0.204	1.468	0.162	24.203	0.096	0.074	3.47	0.506	21.767	0.423
Verbenol	5.811	5.896	0.23	0.189	0	0.222	5.252	0.31	0.502	0.112	0.066	4.186
α-pineno epoxyde	12.857	13.044	0.175	7.933	4.487	0.329	0	0.35	10.55	1.116	0.739	0
t-Butanol	316.743	321.368	3.076	313.607	0	0	0	3.185	318.219	0	0	0
Water	245.109	248.945	2.463	242.643	0.007	0	0	2.511	246.427	0	0	0
Acetone	751.625	751.625	7.488	744.033	0	0	0	7.521	744.04	0	0	0
Linalool	8.864	0	0.127	0.502	0.002	6.763	1.373	0	0	0	0	0
2-Methylenebicyclo[2.1.1]_hexane	0	2.25	0	0	0	0	0	0.052	2.175	0	0	0
cis-Pineno-3-ol	0	0.823	0	0	0	0	0	0	0.066	0.015	0.137	0.444
1-Cyclohexene-1-acetaldehydo,α,2-dimethyl	0	2.251	0	0	0	0	0	0.329	0.634	0.035	1.516	0.027
Pinocarvone	5.524	0.658	0.018	0.979	0.51	3.705	0	0.157	0.204	0.01	0.4	0
Myrtenal	5.237	0.827	0.232	1.468	0.946	2.707	0	0	0.496	0.011	0.397	0
Menthene	4	0	0	3.152	0.735	0.001	0	0	0	0	0	0
Isocamphane	2.975	3.982	0.036	2.462	0.31	0	0	0.199	3.742	0.093	0.004	0
α-Fenchene	2.341	3.111	0.071	2.219	0.297	0	0	0	3.147	0.083	0.007	0
α-Farnesene	10.348	0	0.133	0.666	0.002	3.781	5.723	0	0	0	0	0
Lynalyl isobutirate	37.077	21.725	0.447	0.731	0.005	0.048	38.99	0.024	0.74	0.424	1.682	20.725

Table S5. Inventory data for the processes considered in the production of carvone in the conditioning zone.

	Input (kg)	Output (kg)	
**Compound**	**T-102**	**T-102 D**	**T-102 B**
d-Limonene	343.9576	15.0427	328.8973
β-Myrcene	9.0357	1.2594	0
α-Pinene	4.2842	2.3966	0
Sylvestrene	2.7359	0.2961	2.4518

Table S6. Inventory data for the processes considered in the production of carvone in the reaction zone.

	R-101			
	Input (kg)		Output (kg)	
**Compound**	**SI-raw-oils**	**SII-purified-oils**	**SI-raw-oils**	**SII-purified-oils**
Catalyst	12.5	12.5	12.5	12.5
d-Limonene	194.9033	194.9035	34.2032	70.8112
TBHP	465.8383	465.8384	99.5103	76.5162
β-Myrcene	5.1201	0	0.7258	0
α-Pinene	2.4276	0	0.2968	0
Sylvestrene	1.5503	1.4429	0.2613	0.2383
Carvona	0	0	13.033	10.5165
Carveol	0	0	5.2464	4.2334
1,2-Limonene epoxyde	0	0	3.787	3.9759
t-Butanol	0	0	395.9017	396.6785
Water	203.2542	203.2542	284.9672	275.0057
Acetone	751.6249	751.6105	751.6248	751.6246
p-Cresol	0	0	2.2581	1.8069
m-Menth-1(7),8-diene	0	0	0.4041	0.8989
1,6-Dimethyl hept-1,3,5-triene	0	0	0.697	0.7721
p-Menth 2,8-diene-1-ol	0	0	1.272	0.8549
p-Menth 1(7),8-diene-2-hydroperoxyde	0	0	2.4171	2.126
1,3,3-trimethyl-2-vinyl cyclohexene	0	0	7.1114	5.612
α-Farnesene	0	0	14.0354	10.7891
β-Bergamotene	0	0	6.8874	4.5334

Table S7. Inventory data for the processes considered in the production of carvone in the separation zone.

	Input (kg)		Output (kg)									
	SI-raw-oils	SII-purified-oils	SI-raw-oils					SII-purified-oils				
**Compound**	**P-101**		**F-101 S**	**D-101 D**	**T-101 D1**	**T-101 D2**	**T-101 B**	**F-101 S**	**D-101**	**T-101 D1**	**T-101 D2**	**T-101 B**
Catalyst	12.5	12.5	12.5	0	0	0	0	12.5	0	0	0	0
d-Limonene	34.2032	70.8112	0.3755	16.2093	15.681	1.7384	0	0.493	9.4479	56.7401	2.5524	0
TBHP	99.5103	76.5162	1.0544	96.1836	2.3459	0	0	0.7736	42.3267	33.5965	0	0
β-Myrcene	0.7258	0	0.0058	0.5768	0.1425	0.0004	0	0	0	0	0	0
α-Pinene	0.2968	0	0.003	0.2174	0.0753	0.0005	0	0	0	0	0	0
Sylvestrene	0.2613	0.2383	0.0027	0.1454	0.1049	0.0056	0	0.0022	0.0425	0.2125	0.0008	0
Carvona	13.033	10.5165	0.2448	0.9988	0.0712	11.179	0.0017	0.0389	0.1662	0.5155	8.0408	0

Carveol	5.2464	4.2334	0	0.0948	0.03	3.2735	1.2649	0	0.0123	0.0094	3.0669	0.4275
1,2-Limonene epoxyde	3.787	3.9759	0.0396	1.3584	1.7696	0.5523	0	0.2597	0.3839	3.1344	0.2899	0
t-Butanol	395.9017	396.6785	3.9046	392.1635	0.0015	0	0	4.0381	392.7444	0.0002	0	0
Water	284.9672	275.0057	2.8262	281.8048	0.2998	0	0	2.7583	231.5506	40.7618	0	0
Acetone	751.6248	751.6246	7.5029	744.0571	0	0	0	7.4625	744.0244	0	0	0
p-Cresol	2.2581	1.8069	0.0218	0.2887	0.2476	1.5187	0	0	0.1456	0.506	0.8789	0
m-Menth-1(7),8-diene	0.4041	0.8989	0.004	0.2484	0.1452	0.0049	0	0	0.1868	0.68	0.0009	0
1,6-Dimethyl hept-1,3,5-triene	0.697	0.7721	0.0078	0.603	0.0868	0	0	0	0.3136	0.4575	0	0
p-Menth 2,8-diene-1-ol	1.272	0.8549	0.0138	0.0289	0.006	0.9712	0.1686	0	0.0032	0.0019	0.7174	0.0384
p-Menth 1(7),8-diene-2-hydroperoxyde	2.4171	2.126	0.0242	0.0193	0.0133	0.072	1.8728	0	0.0025	0.0047	0.4447	0.9857
1,3,3-trimethyl-2-vinyl cyclohexene	7.1114	5.612	0.1827	2.0506	3.0218	1.7917	0	0	0.4159	3.9844	0.8018	0
α-Farnesene	14.0354	10.7891	0.2627	0.4012	0.0766	11.9256	0.9264	0.0988	0.0591	0.0238	9.9757	0.0446
β-Bergamotene	6.8874	4.5334	0.0666	0.1523	0.0363	0.0541	6.5985	0.2575	0.0188	0.0099	0.0436	4.4113

Table S8. Inventory of the input energy data for the processes considered in the production of verbenone.

Requirement	Zone	Equipmet	**SI-raw-oils**	**SII-purified-oils**
	Reaction	R-101	-3183.74	-2902.7
		C-101	-13.2558	
		E-102		
		E-103		
		T-101		-92
	Separation	D-101	-754.92	-747.4
Cooling (kW)	Conditioning	T-102		-3.9312
		E-101		
		D-101	766	766
		C-101	10.2367	
	Separation	T-101		43.7
Heating (kW)	Conditioning	T-102		3.9

		P-101	0.0562	0.055
Electrical Power (kW)	Separation	K-101		

Table S9. Inventory of the input energy data for the processes considered in the production of carvone.

Requirement	Zone	Equipmet	**SI-raw-oils**	**SII-purified-oils**
Cooling (kW)	Reaction	R-101	-3183.738	-2902.704
	Separation	C-101	-13.2558	
		E-102		
		E-103		
		T-101		-92
		D-101	-754.92	-747.4
	Conditioning	T-102		-3.9312
Heating (kW)	Separation	E-101		
		D-101	766	766
		C-101	10.2367	
		T-101		43.7
	Conditioning	T-102		3.9
Electrical Power (kW)	Separation	P-101	0.0562	0.055
		K-101		

S4. Transportation

Colombian city of Girardota was selected as plant location due to its proximity to large cities and the availability of suppliers of orange oil and turpentine, **Figure S3**. As the remaining reagents required in the process are not found in the country, they would have to be imported from nearby suppliers such as the USA, the closest available. **Table S10** shows the processes to transport acetone, *t*-butyl hydroperoxide and the catalyst from the production plant to the closest port by diesel truck, then to Colombia by maritime way and finally to Girardota by diesel truck.

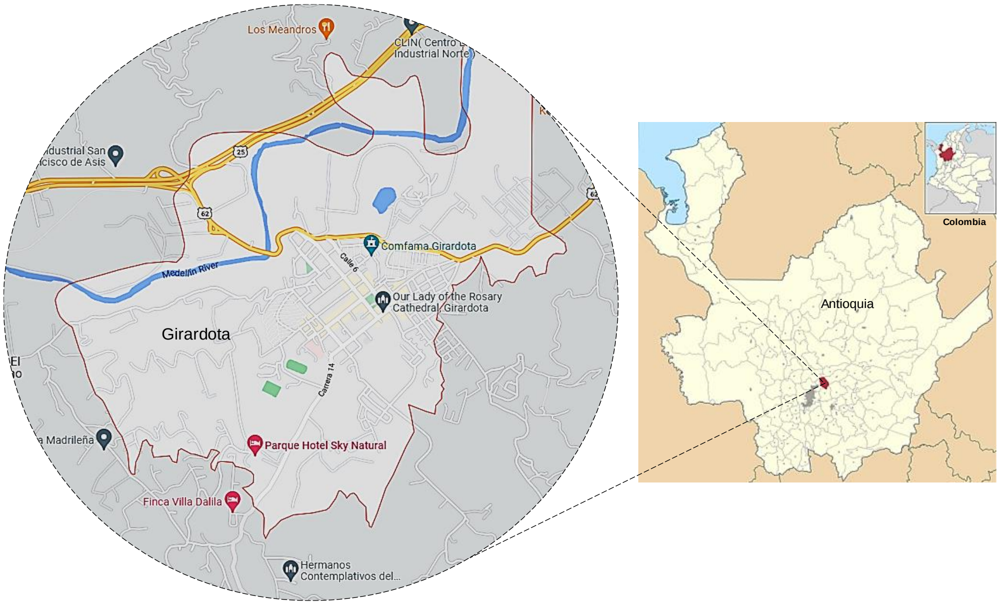

Figure S3. Site location for the batch production of verbenone and carvone from turpentine and orange oils oxidation with TBHP and $FePcCl_{16}$-NH_2-SiO_2.

Table S10. Unit and transport processes selected from the databases in SimaPro for the production of the verbenone and carvone.

Unit process	Function
Acetone, liquid {RoW}	Solvent, synthesis of $FePcCl_{16}$ complex, synthesis of NH_2-SiO_2 support
Allyl chloride {RoW}	Synthesis of allylamine
Aluminium chloride {GLO}	Isobutane isomerization
Ammonia {RoW}	Synthesis of allylamine
Argon, liquid {roW}	Preparation of $FePcCl_{16}$-NH_2-SiO_2 catalyst
Carbon dioxide, liquid {RoW}	Synthesis of ammonium heptamolybdate
Chlorine, gaseous {RoW}	Synthesis of tetrachlorophtalic anhydride
Copper {GLO}	Synthesis of trietoxysilane
Copper oxide {GLO}	Synthesis of trietoxysilane
Dichloromethane {RoW}	Synthesis of $FePcCl_{16}$ complex
Electricity, low voltage {CO}	Isobutane isomerization, verbenone/carvone separation
Ethanol, without water, in 99.77% solution state, from ethylene {RoW}	Synthesis of trietoxysilane, synthesis of $FePcCl_{16}$ complex
Heat, central or small-scale, natural gas {RoW}	Heating in separation and synthesis equipment
Hydrochloric acid, without water, in 30% solution state {RoW}	Isobutane isomerization
Iron(II) chloride {GLO}	Synthesis of $FePcCl_{16}$ complex
Liquefied petroleum gas {RoW}	Isobutane isomerization
Molybdenum {GLO}	Synthesis of tetrachlorophtalic anhydride
Molybdenum trioxide {GLO}	Synthesis of ammonium heptamolybdate
Nitrobenzene {RoW}	Synthesis of FePcCl16 complex
Oxygen, liquid {RoW}	Isobutane isomerization

Phtalic anhydride {GLO}	Synthesis of tetrachlorophtalic anhydride
Rhodium {GLO}	Synthesis of APTES
Sand {roW}	Synthesis of trietoxysilane
Silica fume, densified {GLO}	Synthesis of NH_2-SiO_2 support
Sodium hydroxide, without water, in 50% solution state {GLO}	Synthesis of $FePcCl_{16}$ complex
Sulfuric acid {RoW}	Synthesis of $FePcCl_{16}$ complex
Turpentine {GLO}	Source of turpentine
Urea, as N {GLO}	Synthesis of $FePcCl_{16}$ complex
Water, deionised {RoW}	Synthesis of $FePcCl_{16}$ complex
Xylene {RoW}	Synthesis of APTES, synthesis of NH_2-SiO_2 support

**Reagent**	**Country**	**Transport process**
Acetone	USA	Transport, Single unit truck, short haul, diesel powered, East/tkm/RNA
	International waters	Container ship, technology mix, 27500 dwt pay load capacity GLO
	Colombia	Transport, Truck <10t, EURO5, 80%LF, Default/GLO Mass
TBHP	USA	Transport, Single unit truck, short haul, diesel powered, West/tkm/RNA
	International waters	Container ship, technology mix, 27500 dwt pay load capacity GLO
	Colombia	Transport, Truck <10t, EURO5, 80%LF, Default/GLO Mass
Reagents for catalyst synthesis	USA	Transport, Single unit truck, long haul, diesel powered, East/tkm/RNA
	International waters	Container ship, technology mix, 27500 dwt pay load capacity GLO
	Colombia	Transport, Truck <10t, EURO5, 80%LF, Default/GLO Mass

2. Evaluation of the environmental impacts by ReCiPe Midpoint

Environmental impacts were calculated by the ReCiPe 2016 Midpoint (H) method as shown in **Figure S4** to **Figure S7** for both verbenone and carvone process.

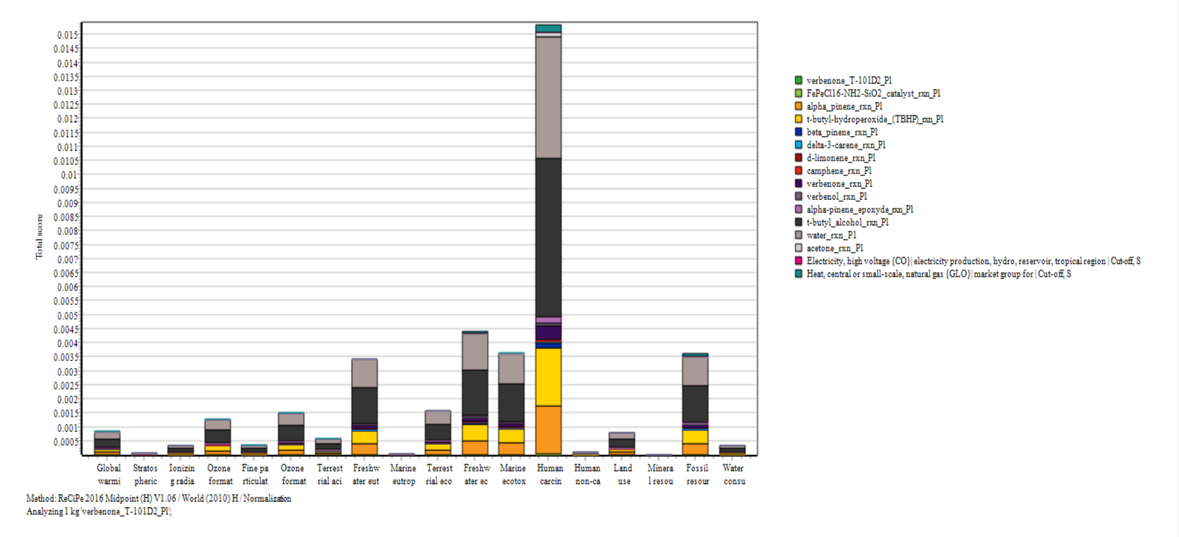

Figure S4. Environmental impact assessment by normalization indicators in the production of verbenone by SI-raw-oils. Assessment method: ReCiPe 2016 Midpoint (H) V1.06 / World (2010) H/A.

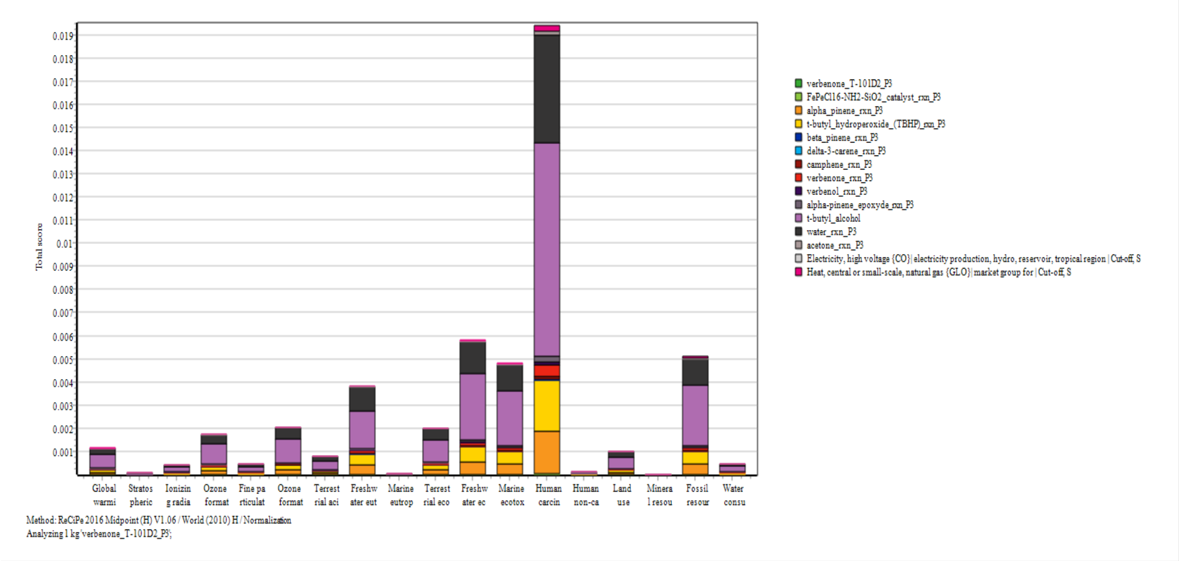

Figure S5. Environmental impact assessment by normalization indicators in the production of verbenone by SII-purified-oils. Assessment method: ReCiPe 2016 Midpoint (H) V1.06 / World (2010) H/A.

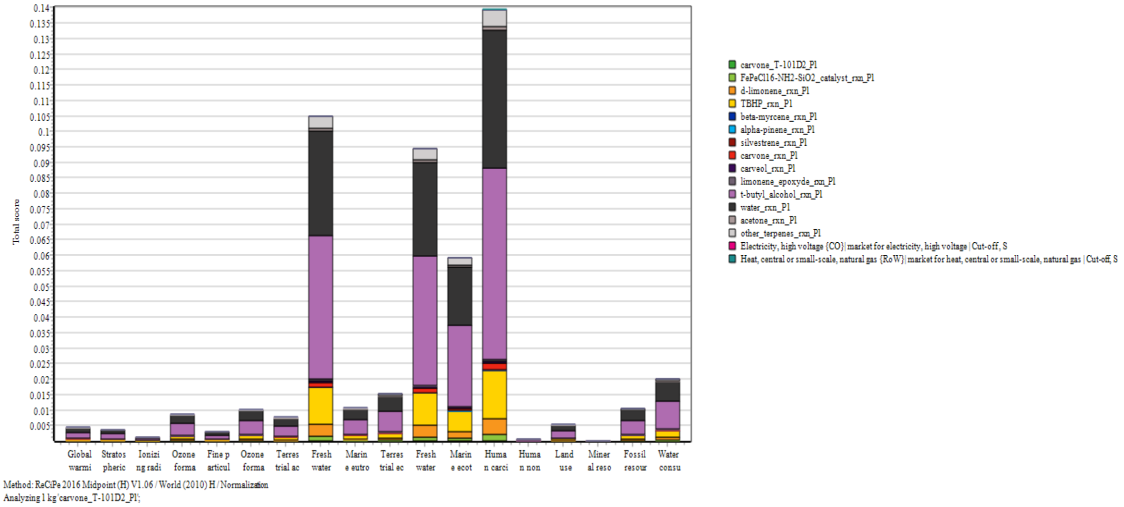

Figure S6. Environmental impact assessment by normalization indicators in the production of carvone by SI-raw-oils. Assessment method: ReCiPe 2016 Midpoint (H) V1.06 / World (2010) H/A.

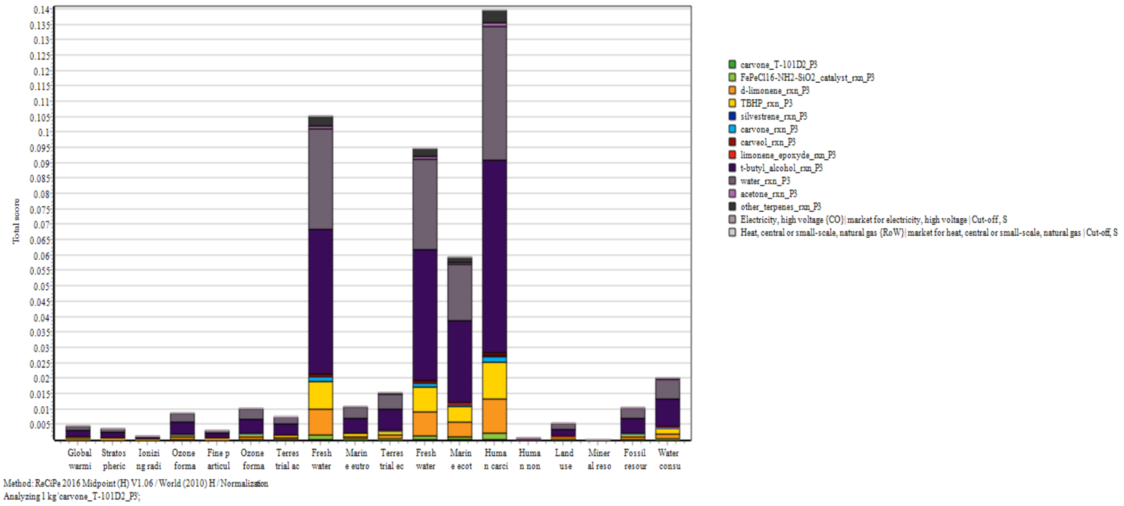

Figure S7. Environmental impact assessment by normalization indicators in the production of carvone by SII-purified-oils. Assessment method: ReCiPe 2016 Midpoint (H) V1.06 / World (2010) H/A.

References

1. Beccali, M.; Cellura, M.; Iudicello, M.; Mistretta, M. Resource Consumption and Environmental Impacts of the Agrofood Sector: Life Cycle Assessment of Italian Citrus-Based Products. *Environ. Manage.* **2009**, *43*, 707–724, doi:10.1007/s00267-008-9251-y.

2. Becerra, J.-A.; Villa, A.-L. Techno-Economic Evaluation of D-Limonene and α-Pinene Separation from Citrus and Turpentine Oils. *Chem. Eng. \& Technol.* **2020**, *43*, 2295–2306,

doi:https://doi.org/10.1002/ceat.201900691.

3. Metz, J.; Schneider, O.; Hanack, M. Synthesis and Properties of Substituted (phthalocyaninato)-Iron and -Cobalt Compounds and Their Pyridine Adducts. *Inorg. Chem.* **1984**, *23*, 1065–1071, doi:10.1021/ic00176a014.

4. Sorokin, A.B.; Tuel, A. Heterogeneous Oxidation of Aromatic Compounds Catalyzed by Metallophthalocyanine Functionalized Silicas . *New J. Chemestry* **1999**, *23*, 473–476.

5. Blume, P.W.; Thomas, G.A.; Gervais, B. Chlorination of Phthalic Anhydride 1974.

6. Ronzio, R.A.; Davis, P.K.; Ziegler, R.C. Process for Producing Ammonium Heptamolybdate And/or Ammonium Dimolybdate 1978.

7. Takatsuna, K.; Shiozawa, K.; Okumura, Y. Process for Preparing Amino-Propyl Silanes 1989.

8. Rösch, L.; John, P.; Reitmeier, R. Silicon Compounds, Organic. In *Ullmann's Encyclopedia of Industrial Chemistry*; John Wiley & Sons, Ltd, 2000 ISBN 9783527306732.

9. Yongsheng, Z.; Renjie, Y.; Chen, J. Production Process of Triethoxy Silane. Jiangyin Jingcheng Glass Co., Ltd., No. CN1810811A 2006, 5.

10. Converse, W. Process for Production of Unsaturated Amines 1940, 4.
